# Bearing Fault Feature Extraction Method Based on Enhanced Differential Product Weighted Morphological Filtering

**DOI:** 10.3390/s22166184

**Published:** 2022-08-18

**Authors:** Xiaoan Yan, Tao Liu, Mengyuan Fu, Maoyou Ye, Minping Jia

**Affiliations:** 1 School of Mechatronics Engineering, Nanjing Forestry University, Nanjing 210037, China; 2 School of Mechanical Engineering, Southeast University, Nanjing 211189, China

**Keywords:** morphological filtering, rolling bearing, feature extraction, fault diagnosis

## Abstract

Aimed at the problem of fault characteristic information bearing vibration signals being easily submerged in some background noise and harmonic interference, a new algorithm named enhanced differential product weighted morphological filtering (EDPWMF) is proposed for bearing fault feature extraction. In this method, an enhanced differential product weighted morphological operator (EDPWO) is first constructed by means of infusing the differential product operation and weighted operation into four basic combination morphological operators. Subsequently, aiming at the disadvantage of the parameter selection of the structuring element (SE) of EDPWO depending on artificial experience, an index named fault feature ratio (FFR) is employed to automatically determine the flat SE length of EDPWO and search for the optimal weighting correlation factors. The fault diagnosis results of simulation signals and experimental bearing fault signals show that the proposed method can effectively extract bearing fault feature information from raw bearing vibration signals containing noise interference. Moreover, the filtering result obtained by the proposed method is better than that of traditional morphological filtering methods (e.g., AVG, STH and EMDF) through comparative analysis. This study provides a reference value for the construction of advanced morphological analysis methods.

## 1. Introduction

The assessment of bearing faults is an essential part of condition monitoring for the pieces of rotating machinery inside a wind turbine [1,2]. If bearing faults cannot be identified and diagnosed in advance, they will easily cause personal injury and economic loss [3,4,5]. Therefore, it is indisputable to explore and develop new fault diagnosis methods. Signal analysis based on vibration is recognized as one of the effective fault diagnosis methods [6,7,8]. However, in actual engineering, when there is a local fault on the surface of a bearing, it will produce a periodic vibration signal with repetitive spikes and multiple harmonic modulation components. At the same time, due to the influence of friction, clearance, stiffness, load and other nonlinear factors, the transient impulse characteristics of bearing vibration signals are comparatively weak and the signal-to-noise ratio (SNR) of internal noise is quite low, which makes it clear that it is difficult to detect rolling bearing damage [9,10]. When traditional digital filtering (e.g., finite impulse response (FIR) filter [11], infinite impulse response (IIR) filter, and Butterworth filter) is utilized to remove the noise of the original bearing vibration signal, the intrinsic characteristics of the original bearing vibration signal may be destroyed due to the cutoff frequency not being set properly. Therefore, research on a new rolling bearing fault feature extraction method is of great research value to prevent bearing damage.

As a nonlinear signal processing method proposed by Serra and Matheron, mathematical morphology (MM) was originally utilized to predict the value of an area’s mineral resources [12,13,14]. Nowadays, this method has been widely used in the fault feature extraction of mechanical equipment. For instance, Shen et al. [15] constructed a new morphological filtering system by using the combination of morphological opening–closing operations and closing–opening operations and adopted the local extremum of the signal to determine the length of the structuring element (SE) of morphological filtering. Hao and Chu [16] utilized a morphological undecimated wavelet decomposition method to process bearing vibration data and obtain an effective fault diagnosis result. Li et al. [17] constructed a new morphological filtering method called the weighted multi-scale morphological gradient operator to detect fault signatures hidden in the raw bearing vibration data and achieve effective bearing fault detection. Dong et al. [18] proposed a morphological filter named the combination average operator (AVG) of the closing and opening operations to extract periodic impulse components and used signal-to-noise ratio (SNR) to determine its SE length. Yan and Jia [19] proposed a novel morphological operator called the combination morphological-hat transform for conducting the effective fault feature extraction of wind turbine vibration signals and used the particle swarm optimization (PSO) algorithm to adaptively determine the length of the SE of the proposed method. Raj et al. [20] proposed a morphological self-complementary top-hat (STH) method to extract bearing fault features and achieved a good bearing fault diagnosis result. Zhang et al. [21] proposed a method named multi-scale morphological difference filtering to extract fault features from the collected bearing vibration data, where its SE scale is automatically determined by using the adaptive peak search method. Li and Liang [22] constructed a continuous scale mathematical morphology method and integrated the frequency-domain kurtosis criterion to select the component located in the optimal scale band to extract impulsive features and distinguish bearing fault type. Osman and Wang [23] first presented a new technique named morphological Hilbert–Huang transform based on closing–opening differential filtering, and then adopted an integration index based on kurtosis with Renyi entropy to select the optimal SE scale of the proposed method to pre-process bearing vibration signals. Meanwhile, empirical mode decomposition (EMD) is applied to separate the filtering signal into several intrinsic mode components. Finally, spectrum analysis of intrinsic mode components is conducted to identify bearing faults. Cui et al. [24] proposed a novel multi-scale morphological filtering algorithm based on the information-entropy threshold (IET-MMF) to effectively extract fault feature information and implement early fault detection of rolling bearings. Luo et al. [25] proposed a bearing fault diagnosis method based on enhanced morphological difference filtering (EMDF) to detect bearing faults, and the effectiveness of the proposed method is demonstrated by experimental comparison results. Hu et al. [26] proposed an improved morphological filtering algorithm to extract fault feature information from the bearing vibration signal with low SNR. Chen et al. [27] presented an adaptive time-varying morphological filter (ATVMF) to extract bearing fault information and improve fault feature extraction capabilities. Tang et al. [28] proposed an unbiased-autocorrelation morphological filter (UAMF) to remove random impulse interference by combining a morphological filter with an autoregressive filter, and the effectiveness of the algorithm is demonstrated by the simulation and experiment analysis. Although the aforementioned morphological filtering methods are successfully applied in fault detection, they still have some problems. Firstly, there is no clear guide for the selection of the SE scale of morphological operators. Secondly, the calculation process of most morphological filtering is complicated for bearing vibration signals under a heavy noise environment, and the elimination of in-band noise of bearing vibration signals is not complete due to the improper SE parameter settings. That is, the universality of traditional morphological filtering is not strong.

Considering the above-mentioned situations, to improve bearing fault feature extraction ability and avoid the problem of empirical selection of parameters of morphological filtering, this paper proposed a bearing fault feature extraction method based on enhanced differential product weighted morphological filtering (EDPWMF), where its combination parameters can be automatically determined by using fault feature ratio (FFR). The effectiveness of the proposed method for bearing fault feature extraction is demonstrated by simulation analysis and two experimental case studies. Moreover, quantitative comparative analysis is also performed to validate the superiority of the proposed method.

The rest of this paper is organized as follows: Section 2 briefly introduces the theoretical background of morphological filtering. Section 3 elaborates the contents of the proposed method in detail, including the construction and adaptive parameter selection of EDPWO. Simulation analysis and experimental verification are conducted in Section 4 and Section 5, respectively, including the comparative analysis and discussion of results. Finally, the conclusion is summarized in Section 6.

## 2. Theory Background

Morphological filtering is a nonlinear filtering method based on mathematical morphology theory, which can effectively eliminate noise and retain some shape feature information in the original bearing vibration signal. The basic principle of applying morphological filtering for signal analysis is to use a structuring element (SE) with a specific shape to detect the feature information of a signal. Four common morphological operators include dilation, erosion and opening and closing operations [29].

Assuming that f(n),n=1,2,⋯,N is a discrete one-dimensional time series, g(m),m =1,2,⋯,M is a unit SE, and the length *M* of SE is less than that of f(n). The dilation operation and erosion operation of f(n) with respect to g(m) are respectively defined as:

(1) (f⊕g)(n)=max{f(n−m)+g(m)}

(2) (f⊖g)(n)=min{f(n−m)−g(m)}

The opening operation and closing operation are essentially the combined results of the dilation and erosion operation. The opening operation and closing operation are respectively defined as:

(3) (x∘g)(n)=(x⊖g⊕g)(n)

(4) (x·g)(n)=(x⊕g⊖g)(n)

where ⊕ is the dilation operator, ⊖ is the erosion operator, ∘ is the opening operator, and · is the closing operator.

The erosion operation can filter out unnecessary noise, smooth the negative impulse of a signal, and eliminate the positive impulse of a signal. The dilation operation has the opposite effect on the signal as the erosion operation. Similarly, the function of the opening operation is to filter the peak noise of a signal and remove edge burr of the signal. In contrast to the opening operation, the closing operation can be utilized to smooth or suppress the noise in the trough of the signal and fill in the holes and gaps in the signal.

## 3. The Proposed Method

### 3.1. Construction of EDPWO

To ensure that the shape characteristics of the original bearing vibration signal are not damaged, the closing operation and dilation operation are combined to enhance the feature extraction ability of the morphological operator, which can suppress the negative impulse and retain or expand the positive impulse. However, considering that the combination morphological operator may damage the negative impulse, it is necessary to conduct the erosion operation after the closing and dilation operations, so that the negative impulse in the bearing vibration signal can be amended. Therefore, two combinations morphological operators can be defined as follows:

(5) $$F_{CDE}\left(f\left(n\right)\right)=\left(f\cdot g⊕g⊖g\right)(n)$$

(6) $$F_{DCE}\left(f\left(n\right)\right)=\left(f⊕g\cdot g⊖g\right)(n)$$

Similarly, the feature extraction ability of the morphological operator is enhanced by combining the opening operation and erosion operation, which can suppress the positive impulse and retain or enlarge the negative impulse. However, considering that the combination operator may destroy the positive impulse, it is necessary to conduct the dilation operation after the opening and erosion operations, so that the positive impulse in the bearing vibration signal can be amended. Therefore, another two combinations of morphological operators can be defined as follows:

(7) $$F_{EOD}\left(f\left(n\right)\right)=\left(f⊖g\circ g⊕g\right)(n)$$

(8) $$F_{OED}\left(f\left(n\right)\right)=\left(f\circ g⊖g⊕g\right)(n)$$

To further extract the positive and negative impulses from the bearing vibration signal simultaneously, two new weighted synthetic morphological operators ($F_{CDE\&OED}$ and $F_{DCE\&EOD}$) are proposed by adding the weight coefficients into the above combination operators, which are aimed at achieving the optimal feature extraction performance. The two new weighted synthetic morphological operators can be defined as follows:

(9) $$F_{CDE\&OED}(f(n))=\mu_{1}F_{CDE}(f(n))-\mu_{2}F_{OED}(f(n))$$

(10) $$F_{DCE\&EOD}(f(n))=\mu_{1}F_{DCE}(f(n))-\mu_{2}F_{EOD}(f(n))$$

where $\mu_{1}$ and $\mu_{2}$ are the weighting coefficients of ($F_{CDE}$ or $F_{DCE}$) and ($F_{OED}$ or $F_{EOD}$), respectively. The weighted synthetic morphological operator can not only reduce the noise interference, but also avoid the problem of deviation of the filtered signal. When the weighting coefficients are calculated, they should satisfies the following formula:

(11) $$\{\begin{array}{c} \mu_{1}=r_{1}/r_{2}+r_{1}\\ \mu_{2}=r_{2}/r_{1}+r_{2} \end{array}$$

where $r_{1}$ and $r_{2}$ are two weighting correlation factors, which can be determined by using some sensitive indicators. To maximize the ability of bearing fault feature extraction, in this paper, based on $F_{CDE\&OED}$ and $F_{DCE\&EOD}$ operators, an enhanced differential product weighted morphological operator (EDPWO) is proposed, which is defined as follows:

(12) $$\mathrm{EDPWO}=F_{CDE\&OED}(f(n))*F_{DCE\&EOD}(f(n))$$

Theoretically, if $F_{CDE\&OED}$ and $F_{DCE\&EOD}$ can suppress noise interference and extract impulsiveness fault characteristic, EDPWO also can suppress noise interference and even enhance bearing fault feature information.

### 3.2. Adaptive Parameter Selection of EDPWO

Relevant studies have shown that the selection of type and size of the structuring element (SE) is largely related to the analysis performance of morphological filtering. Due to the fact that the flat SE has the advantages of simple structure and fast operation, this paper intends to adopt the flat SE for analysis. The length is the main parameter of the flat SE. If the length of flat SE is very short, morphological operators can extract more impulse features from the bearing vibration signal, but will retain a lot of noise interference and make it difficult to extract bearing fault features. On the contrary, if the length of the flat SE is too long, some useful fault information will be filtered out and only a few impulse features can be extracted. Therefore, the selection of the length of the flat SE has a great influence on the performance of the morphological operator.

Fault feature ratio (FFR) has been proven to be an effective sensitive index in describing bearing fault information [30,31]. For a signal *x*(*t*), the FFR can be defined as:

(13) $$\mathrm{FFR}=\frac{A_{1}(f)+A_{2}(f)+A_{3}(f)}{\sum_{i=1}^{n}A_{n}(f)}$$

where $A_{n}(f)$ is the amplitude corresponding to all frequencies in whole envelope spectrum of the signal *x*(*t*). $A_{1}(f)$, $A_{2}(f)$ and $A_{3}(f)$ are the amplitudes corresponding to the first three harmonics of bearing fault characteristic frequency in the envelope spectrum of the signal *x*(*t*), respectively. Generally speaking, the higher FFR is, the better the effect of morphological filtering is, and it is more advantageous to extract bearing fault characteristics. Therefore, in this paper, FFR is employed to carry out adaptive selection of the length of flat SE of the proposed EDPWO. Meanwhile, the weighting correlation factors $r_{1}$ and $r_{2}$ of EDPWO are also adaptively selected by iterative operation with the help of FFR.

### 3.3. The Proposed Method

**Figure 1:**
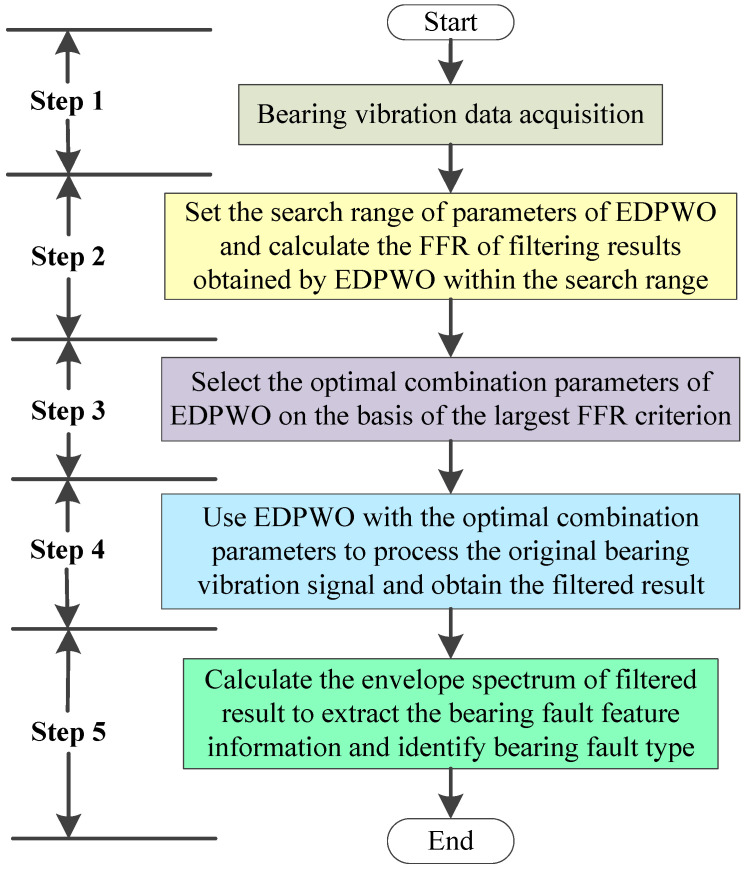
The flow chart of the proposed method for bearing fault feature extraction.

In order to obtain useful bearing fault feature information and improve fault diagnosis accuracy, a novel approach based on enhanced differential product weighted morphological filtering (EDPWMF) is proposed for bearing fault feature extraction. Figure 1 shows the flow chart of the proposed method, and the specific process is as follows:

**Step 1:** The bearing vibration signal is collected by installing the sensors on the mechanical fault simulator.

**Step 2:** Set the search range of parameters (i.e., the length of flat SE, the weighting correlation factors $r_{1}$ and $r_{2}$) of EDPWO, and calculate the FFR of filtering results obtained by EDPWO for the raw bearing vibration signal within the search range.

**Step 3:** The optimal combination parameters (i.e., the optimal flat SE length *M*, the optimal weighting correlation factors $r_{1}$ and $r_{2}$) of EDPWO are selected on the basis of the largest FFR criterion.

**Step 4:** Use EDPWO with the optimal combination parameters to process the original bearing vibration signal and obtain the filtered result.

**Step 5:** Calculate the envelope spectrum of filtered result to extract the bearing fault feature information and identify bearing fault type.

## 4. Simulation Analysis

### 4.1. Simulation Signal Model

To study the effectiveness of the proposed method, the bearing fault simulation signal *x*(*t*) is constructed as follows:

(14) $$\left\{ \begin{array}{l} x(t)=awgn(s(t),\mathrm{SNR})+y(t)\\ s(t)=\sum_{i}A_{i}h(t-iT-\tau_{i})\\ h(t)=\exp(-Ct)\sin(2\pi f_{n}t)\\ A_{i}=A_{0}\sin(2\pi f_{r}t) \end{array}\right.$$

where *x*(*t*) is composed of three parts (i.e., periodic impulse signal *s*(*t*), sinusoidal harmonic interference signal *y*(*t*) and Gaussian white noise). The harmonic interference signal *y*(*t*) is composed of two sinusoidal waves sin(40πt) and sin(60πt). According to the literature [31], the Gaussian white noise is added into the original periodic impulse signal *s*(*t*) by using MATLAB function awgn(s(t),SNR), where SNR = 0 dB. In Equation (14), $A_{0}$ is set as 2, the rotating frequency $f_{r}$ is set as 20 Hz, the attenuation coefficient *C* is 800, the resonance frequency $f_{n}$ is 4000 Hz, the bearing fault characteristic frequency $f_{a}$ = 1/*T* = 110 Hz, $t_{i}$ represents the random fluctuation of the *i*-th shock relative to the period *T*, and it follows normal distribution with the mean value of 0 and the standard deviation of $0.5\%\times f_{r}$. The sampling frequency $f_{s}$ and sampling number are set as 12,000 Hz and 12,000 points, respectively.

**Figure 2:**
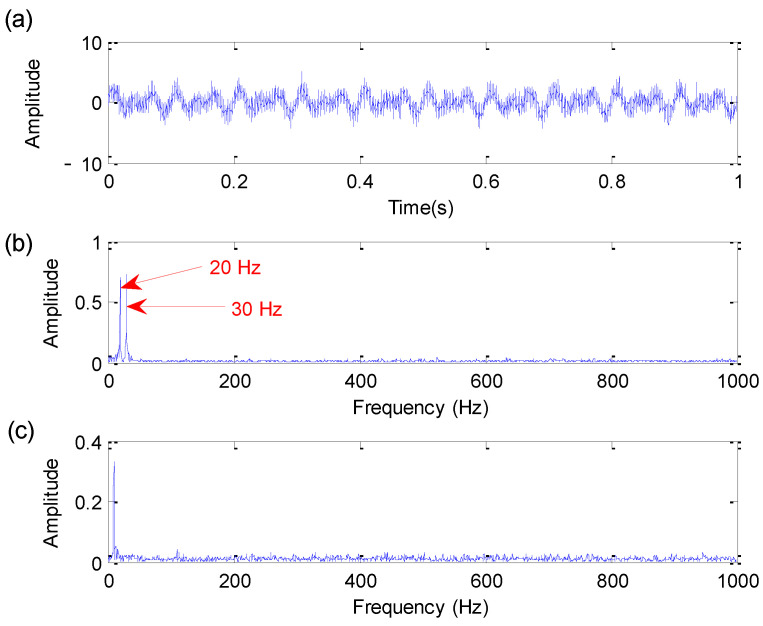
Bearing fault simulation signal: (**a**) Time domain waveform; (**b**) amplitude spectrum; (**c**) envelope spectrum.

Figure 2 shows the time domain waveform, amplitude spectrum and envelope spectrum of the bearing fault simulation signal. As shown in Figure 2, only harmonic interference components of 20 Hz and 30 Hz can be extracted from the amplitude spectrum. Moreover, in the envelope spectrum of Figure 2, bearing fault characteristic frequency $f_{a}$ = 110 Hz is not obvious due to the effect of noise and harmonic interference, which means that it is difficult to extract fault information by direct spectral analysis. 

### 4.2. The Analysis Results of The Proposed Method

**Figure 3:**
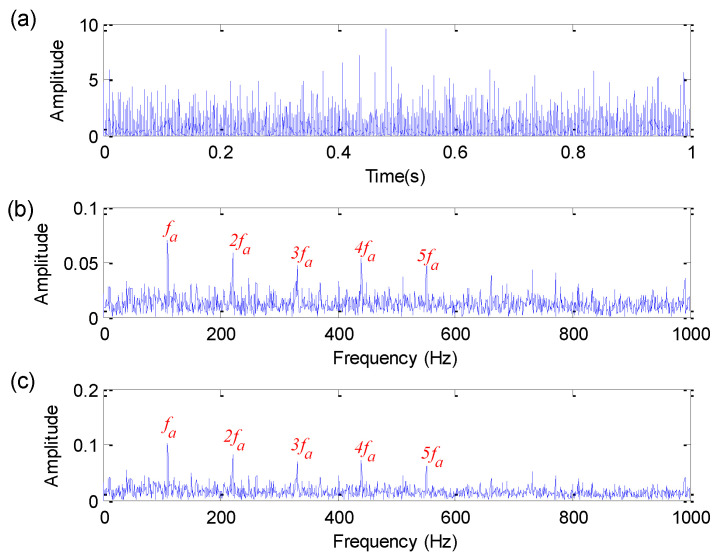
Analysis results obtained by the proposed method for simulation signal: (**a**) Time domain waveform; (**b**) amplitude spectrum; (**c**) envelope spectrum.

The proposed method is utilized to analyze bearing fault simulation signals. Firstly, set the search range of parameters (i.e., the length of flat SE, the weighting correlation factors $r_{1}$ and $r_{2}$) of EDPWO is all set as 1 to 50. Then, the FFR is employed to determine adaptively the optimal combination parameters of EDPWO. Specifically, in EDPWO, the optimal flat SE length *M*, the optimal weighting correlation factors $r_{1}$ and $r_{2}$ are selected as 3, 9 and 10, respectively. Finally, EDPWO with the optimal combination of parameters is used to process bearing fault simulation signals. Figure 3 shows the time domain waveform, amplitude spectrum and envelope spectrum of the filtered signal obtained by the proposed method. It is obvious from the amplitude spectrum and envelope spectrum of Figure 3 that the harmonic interference frequencies of 20 Hz and 30 Hz are effectively removed and the noise is suppressed. Meanwhile, in the amplitude spectrum and envelope spectrum, bearing fault characteristic frequency $f_{a}$ = 110 Hz and its harmonics ($2f_{a}$, $3f_{a}$, $4f_{a}$ and $5f_{a}$) is prominent, which indicates that the proposed method can be used to accurately identify bearing fault information.

**Figure 4:**
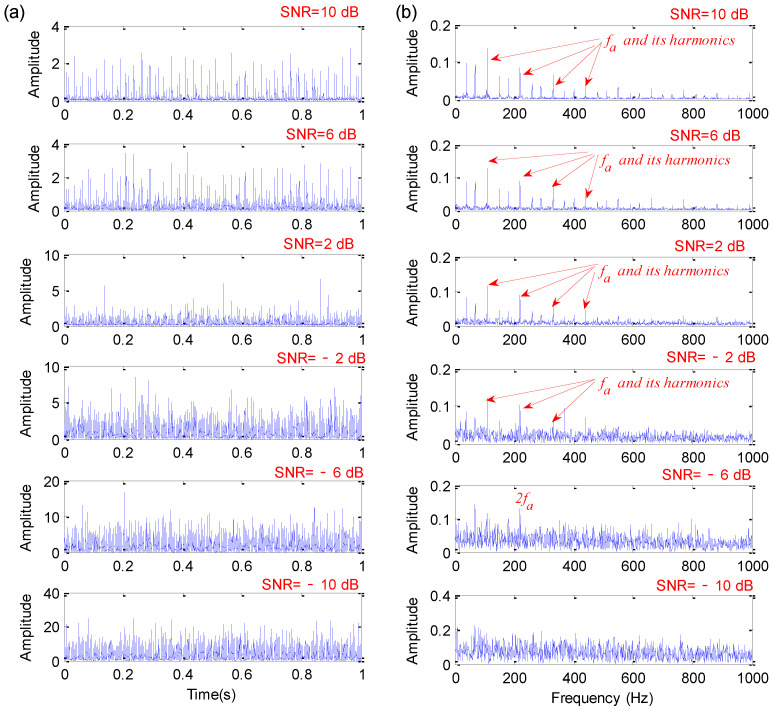
Qualitative analysis results obtained by the proposed method with six levels of noise: (**a**) Time domain waveform; (**b**) envelope spectrum.

To investigate the robustness of the proposed method on noises, the feature extraction capability of the proposed method with increasing levels of noise is systematically considered. Concretely, with the help of the MATLAB function awgn(⋅), the Gaussian white noise is orderly added into the original signal when SNR is decreased from 10 dB to −10 dB. Due to space limitation, Figure 4 only shows the qualitative analysis results obtained by the proposed method with six levels of noise (i.e., SNR = 10 dB, 6 dB, 2 dB, −2 dB, −6 dB, −10 dB). As can be seen from Figure 4, the feature extraction capabilities of the proposed method decrease with the decrease of SNR added into the bearing fault simulation signal. When the added SNR is greater than −2 dB, the proposed method can obtain a bearing fault characteristic frequency of $f_{a}$ = 110 Hz and its harmonics. When the added SNR is −6 dB, the bearing fault characteristic frequency $f_{a}$ is almost invisible, which means that the proposed method is barely able to work in fault information detection. When the added SNR is −10 dB, the bearing fault characteristic frequency $f_{a}$ is entirely submerged, which means that the proposed method is not working at all and has reached its theoretical limit. 

**Figure 5:**
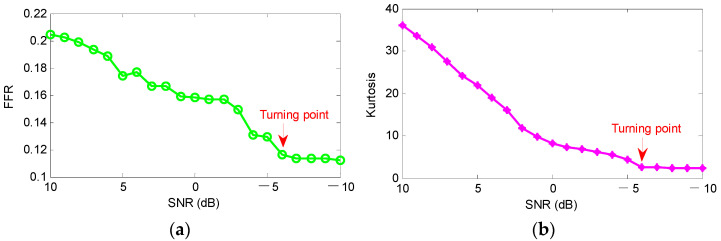
Quantitative analysis results obtained by the proposed method with increasing levels of noise: (**a**) FFR curve; (**b**) kurtosis curve.

To further observe the influence of the added white noise on the effectiveness of the proposed method, we also calculated the quantitative analysis results (i.e., the FFR curve and kurtosis curve) obtained by the proposed method with increasing levels of noise, as shown in Figure 5. It is obvious from Figure 5 that the two indexes (i.e., FFR and kurtosis) obtained by the proposed method have a downward trend when SNR is set as 10 dB to −10 dB. This indicates that the feature extraction capability of the proposed method decreases gradually with the decrease of SNR. Moreover, as shown in Figure 5, there is a turning point at SNR = −6 dB. That is, when SNR is lower than −6 dB, the two indexes (i.e., FFR and kurtosis) obtained by the proposed method gradually remain unchanged, which further indicates that the proposed method will reach its theoretical limit if the added levels of noise become increasingly higher.

### 4.3. Comparisons among Different Methods

**Figure 6:**
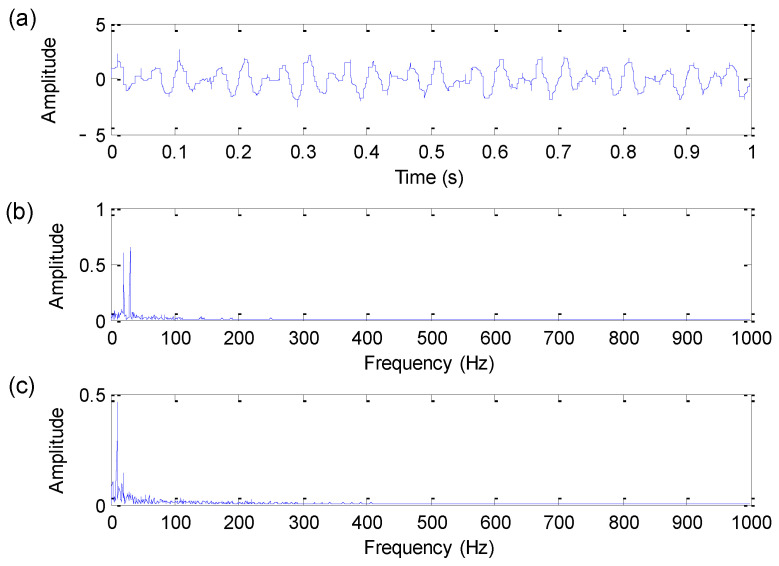
Analysis results obtained by AVG method for simulation signal: (**a**) Time domain waveform; (**b**) amplitude spectrum; (**c**) envelope spectrum.

**Figure 7:**
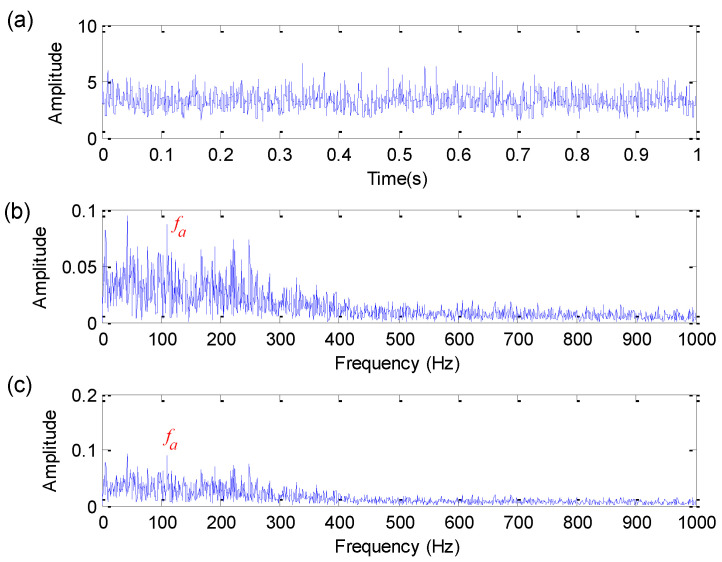
Analysis results obtained by STH method for simulation signal: (**a**) Time domain waveform; (**b**) amplitude spectrum; (**c**) envelope spectrum.

**Figure 8:**
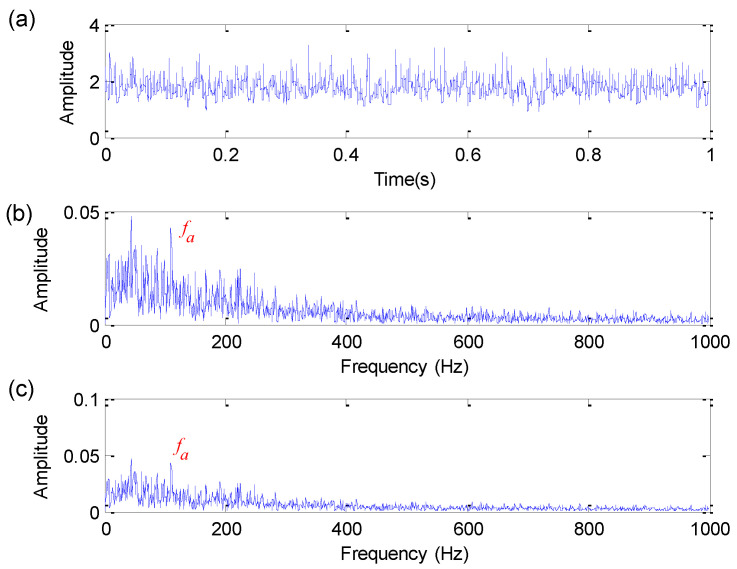
Analysis results obtained by EMDF method for simulation signal: (**a**) Time domain waveform; (**b**) amplitude spectrum; (**c**) envelope spectrum.

To further show the feature extraction ability of the proposed method, the proposed method is compared with three typical morphological operators (i.e., AVG [18], STH [20] and EMDF [25]) and a more traditional non-morphological filter (i.e., Butterworth filter). For the convenience of analysis, this paper takes SNR = 0 dB as an example for comparative analysis. It is worth noting that FFR is used in all three morphological filtering methods to determine the optimal SE parameters. Specifically, the optimal length of flat SE of three methods (i.e., AVG, STH and EMDF) is selected as 21, 5 and 16, respectively. Figure 6, Figure 7 and Figure 8 show the analysis results obtained by AVG, STH and EMDF, respectively. As shown in Figure 6, the bearing fault characteristic frequency $f_{a}$ cannot be found. As can be seen from Figure 7, the bearing fault characteristic frequency $f_{a}$ can be extracted in the amplitude spectrum and envelope spectrum, but its extraction effects are not as good as the proposed method. Similarly, as shown in Figure 8, there is a peak at bearing fault characteristic frequency $f_{a}$, but its harmonics are not obvious. This comparison means that the proposed method has better fault feature extraction performance than three methods (i.e., AVG, STH and EMDF).

**Figure 9:**
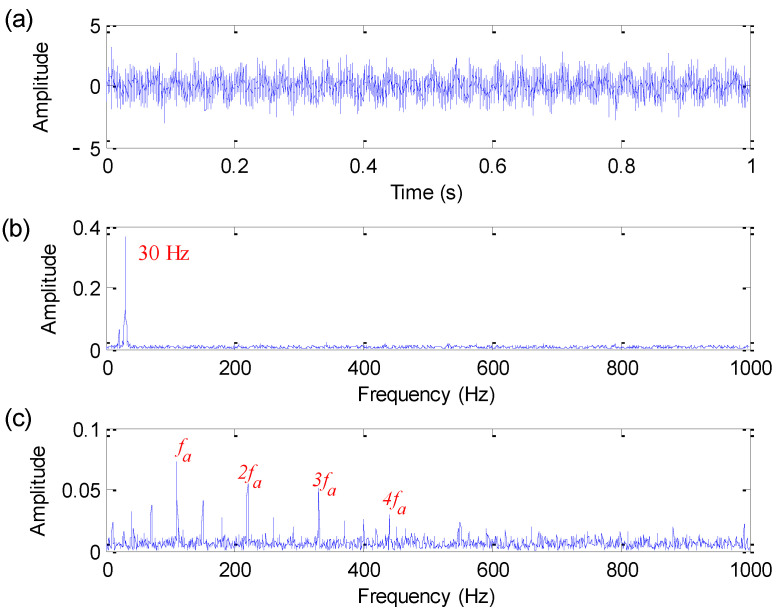
Analysis results obtained by the Butterworth filter for simulation signal: (**a**) Time domain waveform; (**b**) amplitude spectrum; (**c**) envelope spectrum.

**Figure 10:**
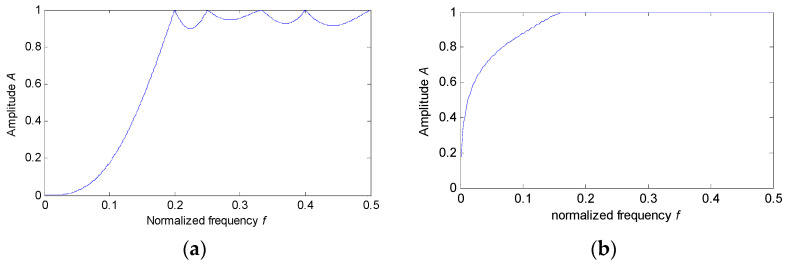
Filtering properties of two methods: (**a**) Proposed EDPWO; (**b**) the Butterworth filter.

Figure 9 shows the analysis results obtained by the Butterworth filter for the simulation signal. Similar to the proposed method, the bearing fault characteristic frequency $f_{a}$ and its harmonics can be extracted obviously in the envelope spectrum of Figure 9c. However, by comparing Figure 3b and Figure 9b, we can find that the harmonic interference component of 30 Hz cannot be removed by using the Butterworth filter to process the simulation signal. This means that the proposed EDPWO can obtain a better feature extraction result than the Butterworth filter, but only from the amplitude spectrum. For a more comprehensive comparison, we will briefly discuss the merits and demerits of two methods (i.e., the proposed EDPWO and the Butterworth filter) from two perspectives. Firstly, from the perspective of filtering properties, according to the single-frequency sinusoidal signal model shown in the previously published literature [32], we calculated the amplitude-versus-frequency curve of the proposed EDPWO, as shown in Figure 10a. Meanwhile, the Butterworth high-pass filter is also designed by using the Fdatool tool in MATLAB, and its amplitude-versus-frequency curve is plotted in Figure 10b. As can be seen from Figure 10, the proposed EDPWO can be categorized as a high-pass morphological operator and has high-pass filtering properties. Compared with EDPWO, the Butterworth filter has steeper amplitude-frequency characteristics before the cutoff frequency and a smaller pass-band ripple. That is, the amplitude-frequency characteristics of the Butterworth filter are better than those of the proposed EDPWO only from the amplitude-versus-frequency curve. Secondly, from the perspective of the practical filtering effect (see Figure 3b and Figure 9b), compared with the Butterworth filter, with the help of SE, the proposed EDPWO can more effectively remove the noise interference without considering the frequency-band distribution. Moreover, due to morphological filtering involving only simple arithmetic operations, compared with the Butterworth filter, the proposed EDPWO has certain competitiveness in running speed.

## 5. Experimental Verification

### 5.1. Case 1: Bearing Data from Laboratory

#### 5.1.1. Experimental Platform and Data Acquisition

**Figure 11:**
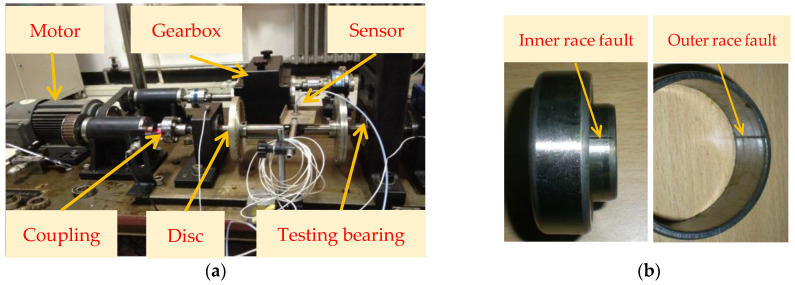
(**a**) Experimental platform; (**b**) the faulty bearing.

In this section, this paper applies experimental bearing fault data from the institute of vibration engineering of North China Electric Power University (NCEPU) to verify the effectiveness of the proposed method. Specifically, the QPZZ-II rotating machinery fault simulation test bench is adopted to collect experimental bearing vibration data induced by the local fault. The photo of the experimental system is illustrated in Figure 11, which is mainly composed of a motor, gearbox, bearing seat, coupling, disc and loader. In the experimental process, Electrical Discharge Machining (EDM) is used to manufacture the scratches on the bearing inner and outer race to simulate the local faults of bearings, respectively. Table 1 lists the dimensions and specifications of the faulty bearings (see Figure 11b). During the experiment, an eddy current sensor with a calibration value of 7.87 V/mm is installed at one position away from the faulty bearing to collect bearing fault data. The motor speed is 1440 r/min, the sampling frequency and sampling number of the signal are 12,800 Hz and 6400 points, respectively. Table 2 displays different bearing fault characteristic frequencies.

#### 5.1.2. Bearing Outer Race Fault Signal Analysis

**Figure 12:**
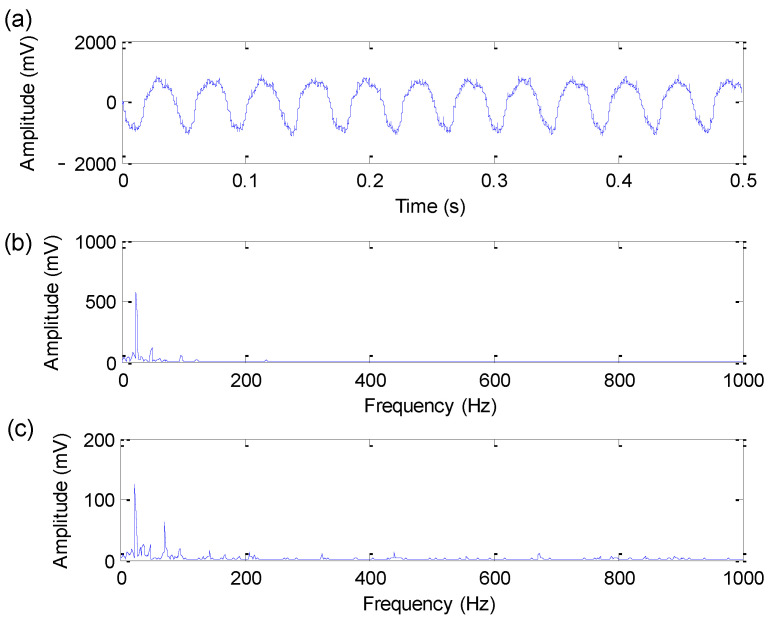
Bearing outer race fault signal: (**a**) Time domain waveform; (**b**) amplitude spectrum; (**c**) envelope spectrum.

**Figure 13:**
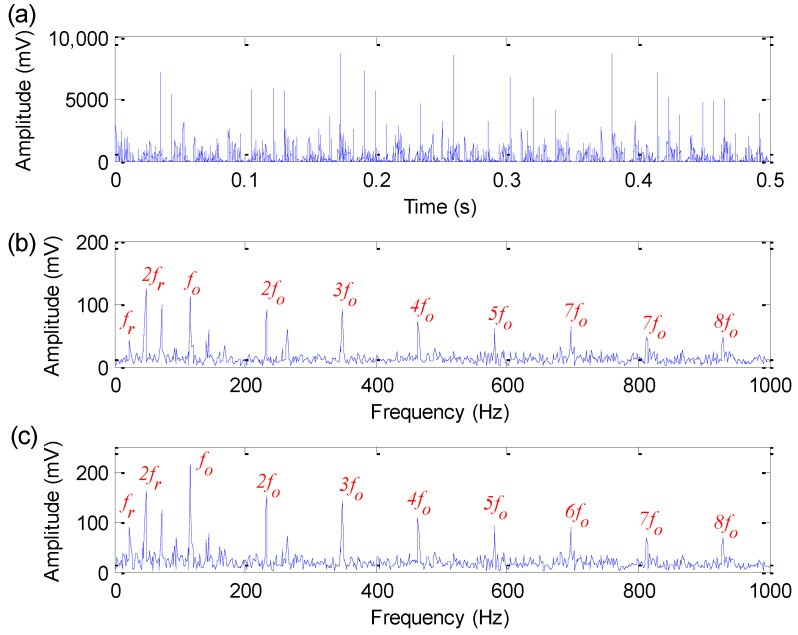
Analysis results obtained by the proposed method for bearing outer race fault signal: (**a**) Time domain waveform; (**b**) amplitude spectrum; (**c**) envelope spectrum.

Figure 12 shows the time-domain waveform, amplitude spectrum and envelope spectrum of the experimental bearing outer race fault signal. In the amplitude spectrum and envelope spectrum shown in Figure 12, the bearing outer race fault characteristic frequency of 115.94 Hz cannot be found. Therefore, to reveal fault information hidden in bearing vibration data, the proposed method is utilized to analyze bearing outer race fault data. Firstly, the search range of parameters (i.e., the length *M* of flat SE, the weighting correlation factors $r_{1}$ and $r_{2}$) of EDPWO is all set as 1 to 50. According to the FFR and iterative search, the optimal combination parameters (i.e., the length *M* of flat SE, the weighting correlation factors $r_{1}$ and $r_{2}$) of EDPWO are automatically selected as $\left(M,r_{1},r_{2})=(23,1,1\right)$ to deal with the experimental bearing outer race fault signal. Figure 13 shows the time-domain waveform, amplitude spectrum and envelope spectrum of the filtered signal obtained by the proposed method for bearing outer race fault signal. From the time-domain waveform in Figure 13, it can be seen that the proposed method can effectively remove the noise interference in the original bearing outer race fault signal. At the same time, it can also be seen from the envelope spectrum of Figure 13 that the proposed method can obviously extract bearing outer race fault characteristic frequency $f_{o}$ = 114 Hz and its harmonics, which verifies the effectiveness of the proposed method in bearing outer race fault feature extraction.

#### 5.1.3. Bearing Inner Race Fault Signal Analysis

**Figure 14:**
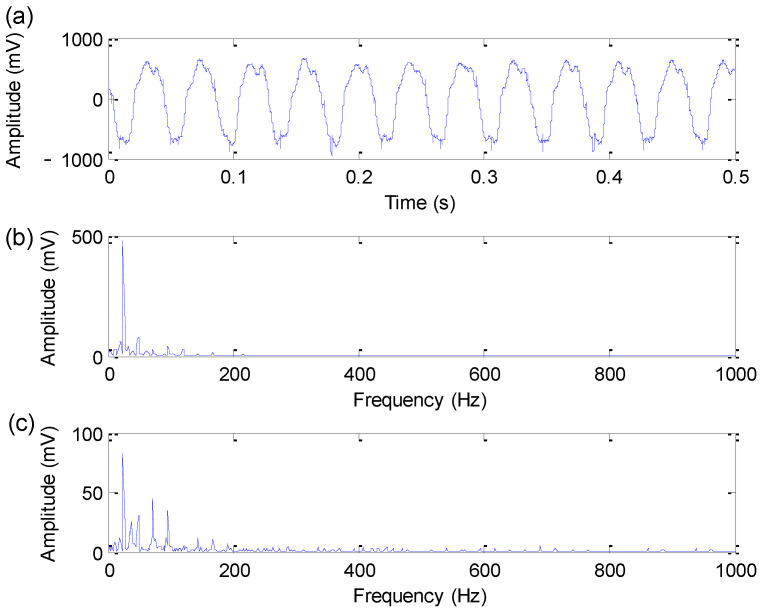
Bearing inner race fault signal: (**a**) Time domain waveform; (**b**) amplitude spectrum; (**c**) envelope spectrum.

Figure 14 shows the time-domain waveform, amplitude spectrum and envelope spectrum of the experimental bearing inner race fault signal. In the amplitude spectrum and envelope spectrum shown in Figure 14, the bearing inner race fault characteristic frequency of 172.09 Hz cannot be extracted. Hence, the proposed method is adopted to analyze the bearing inner race fault signal.

**Figure 15:**
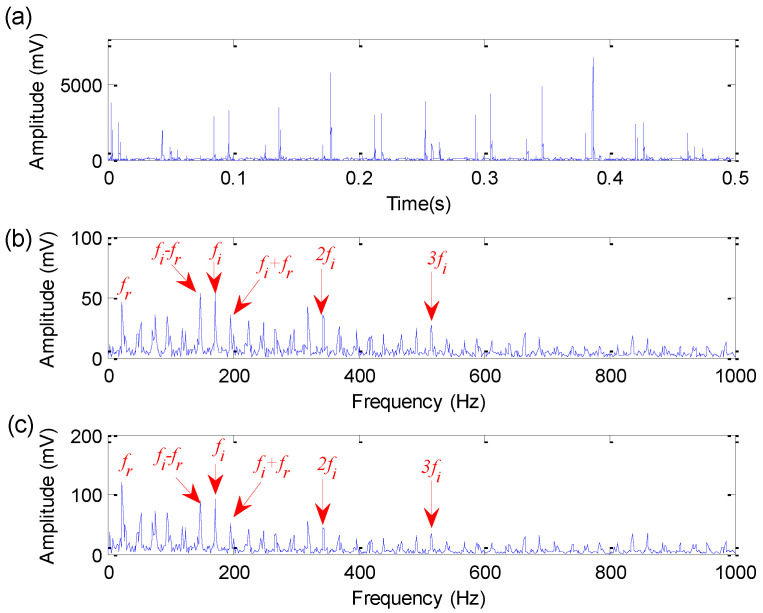
Analysis results obtained by the proposed method for bearing inner race fault signal: (**a**) Time domain waveform; (**b**) amplitude spectrum; (**c**) envelope spectrum.

Firstly, based on the largest FFR criterion and the iterative search operation, the optimal combination parameters $\left(M,r_{1},r_{2}\right)$ of EDPWO are determined as 9, 1 and 5, respectively. Subsequently, EDPWO with the optimal combination of parameters is adopted to process the bearing inner race fault signal. Figure 15 shows the time-domain waveform, amplitude spectrum and envelope spectrum of the filtered signal obtained by the proposed method for bearing inner race fault signal. Seen from the amplitude spectrum and envelope spectrum of Figure 15, bearing inner race fault characteristic frequency $f_{i}$ = 171.9 Hz and its harmonics can be extracted effectively. Meanwhile, the side-band information ($f_{i}-f_{r}$ and $f_{i}+f_{r}$) can also be seen in the amplitude spectrum and envelope spectrum. This indicates that the proposed method is effective in detecting bearing inner race fault information. That is, the effectiveness of the proposed method is demonstrated in bearing inner race fault feature extraction.

#### 5.1.4. Comparison with Several Traditional Morphological Filtering Methods

**Figure 16:**
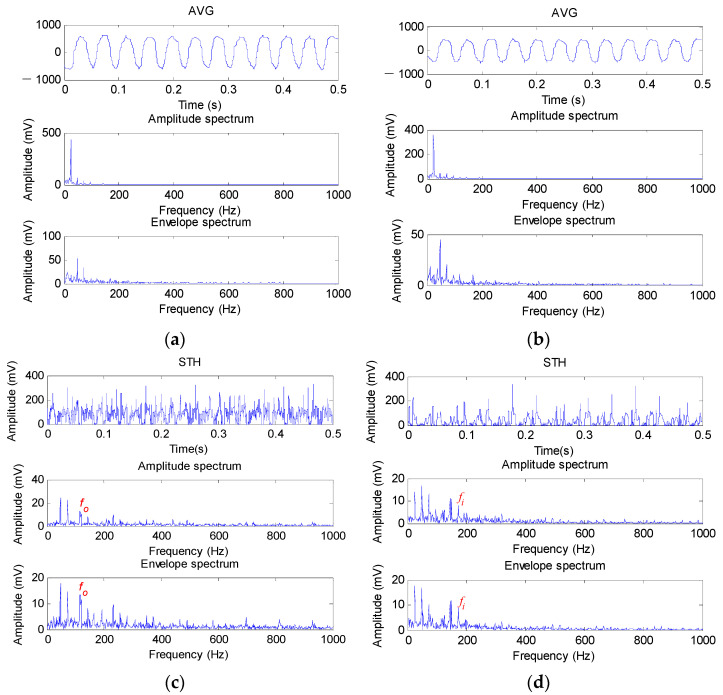

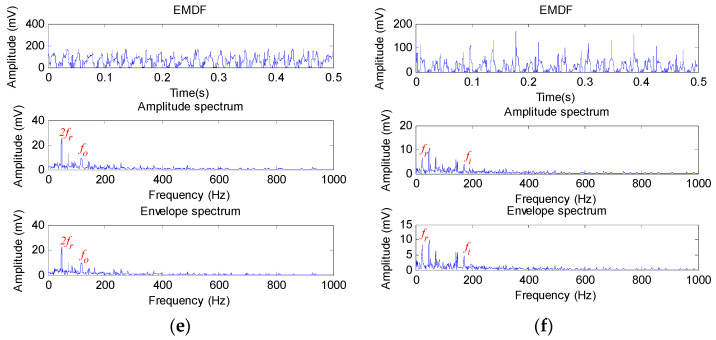
Analysis results obtained by different methods: (**a**) the results of AVG for outer race fault signal; (**b**) the results of AVG for inner race fault signal; (**c**) the results of STH for outer race fault signal; (**d**) the results of STH for inner race fault signal; (**e**) the results of EMDF for outer race fault signal; (**f**) the results of EMDF for inner race fault signal.

In this section, to demonstrate the effectiveness and advantage of the proposed method in bearing fault feature extraction, comparisons between the proposed method and several representative morphological filtering methods (e.g., AVG, STH and EMDF) are conducted. Figure 16a–f show the analysis results obtained by different methods (i.e., AVG, STH and EMDF) for bearing outer race and inner race fault signal, respectively. It can be seen clearly from Figure 16a,b that the bearing outer race and inner race fault characteristic frequencies and their harmonics cannot be found, which indicates that it is difficult to extract bearing fault features by using the AVG operator in this case. It is obvious from Figure 16c,d that the bearing outer race fault characteristic frequency $f_{o}$ and inner race fault characteristic frequency $f_{i}$ can all be extracted in the amplitude spectrum and envelope spectrum of STH, but its harmonics are not as clear as in the proposed method. As can be seen from Figure 16e,f, EMDF can extract bearing outer race fault characteristic frequency $f_{o}$ and inner race fault characteristic frequency $f_{i}$. However, compared with the proposed method, the analysis results of EMDF have two snags: (1) The amplitude of the extracted fault characteristic frequency is not large enough; (2) In the envelope spectrum, the amount of harmonics of fault characteristic frequency is less than that of the proposed method. 

### 5.2. Case 2: Benchmark Data from CWRU

#### 5.2.1. Experimental System Introduction

**Figure 17:**
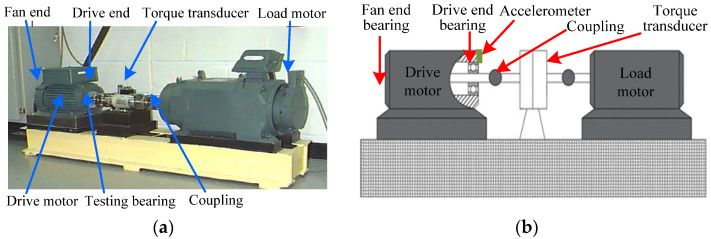
(**a**) The experimental system; (**b**) its structure schematic drawing.

Bearing benchmark data from Case Western Reserve University (CWRU) [33] is introduced to validate the effectiveness of the proposed method. Figure 17 shows the experimental system and its structure schematic drawing, including the drive motor, torque transducer, coupling and load motor. In the course of experimental testing, the local fault is artificially manufactured on a deep groove ball bearing by using electrical discharge machining (EDM). Moreover, one accelerometer is mounted on the bearing seat of the drive end of the motor to collect bearing fault data. Table 3 lists the detailed specifications of the experimental bearing. Table 4 lists the bearing fault characteristic frequency for different loads or motor speeds. In this example, the sampling frequency and sampling number of the signal are set as 12 kHz and 4096 points, respectively.

#### 5.2.2. Statistical Evaluation on All Available Signals

**Figure 18:**
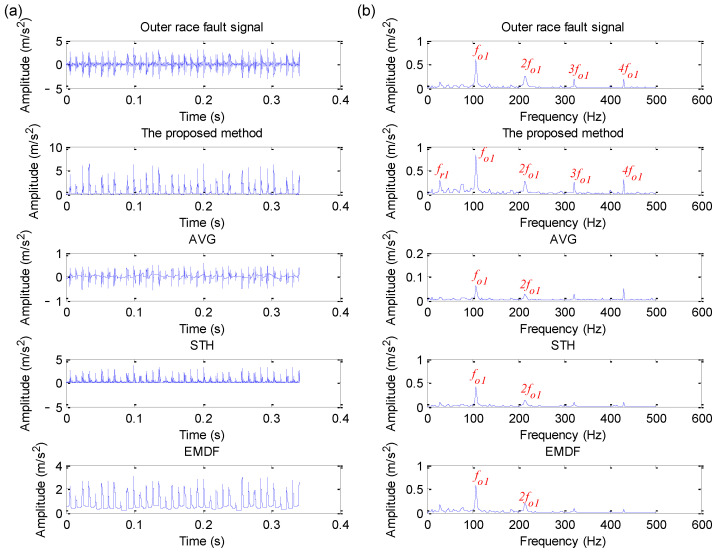
Analysis results obtained by different methods for dataset 130 in case 2: (**a**) Time domain waveform; (**b**) its corresponding envelope spectrum.

**Figure 19:**
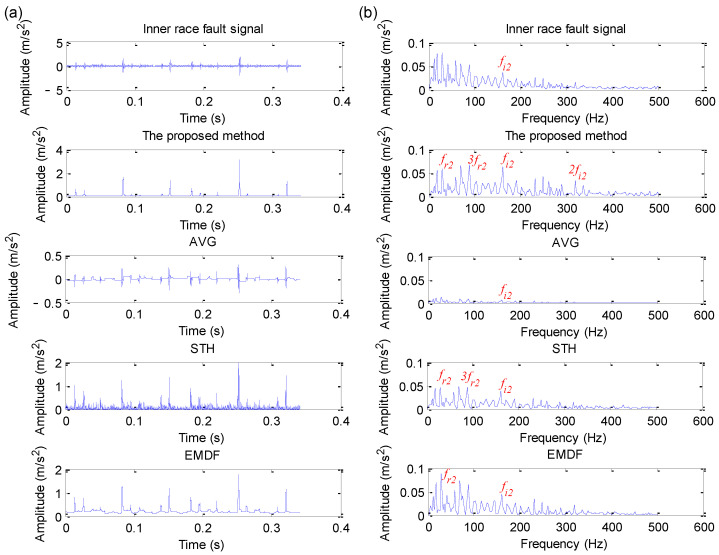
Analysis results obtained by different methods for dataset 170 in case 2: (**a**) Time domain waveform; (**b**) its corresponding envelope spectrum.

**Figure 20:**
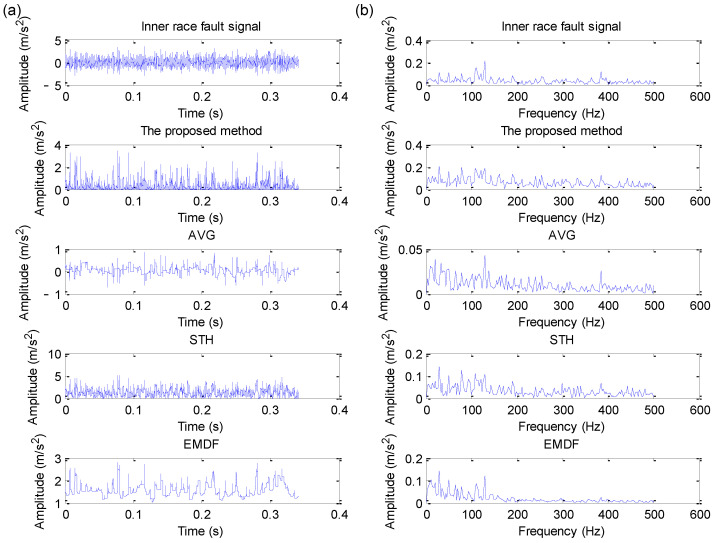
Analysis results obtained by different methods for dataset 3001 in case 2: (**a**) Time domain waveform; (**b**) its corresponding envelope spectrum.

**Figure 21:**
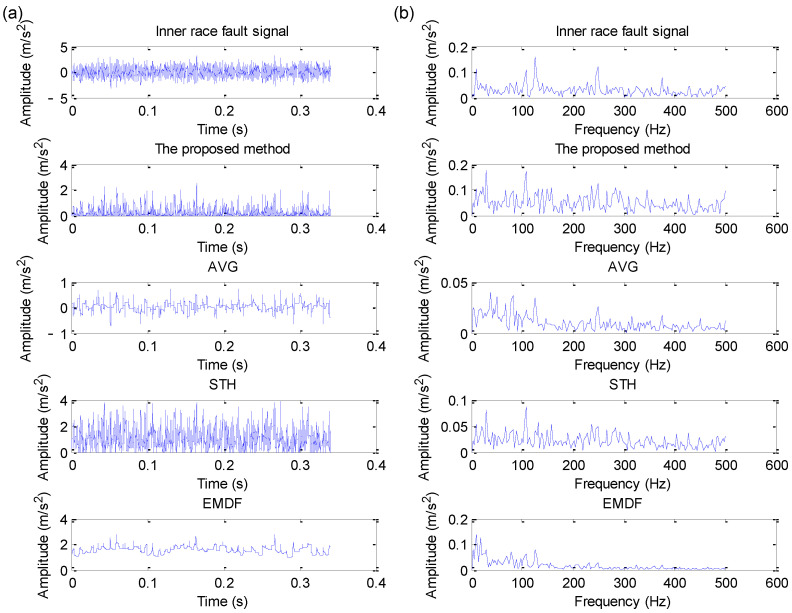
Analysis results obtained by different methods for dataset 3003 in case 2: (**a**) Time domain waveform; (**b**) its corresponding envelope spectrum.

Due to the CWRU benchmark data having different fault data, including four fault diameters and four load values, this section takes the 12 k drive end bearing fault data as an example for statistical analysis. Due to space limitations, this paper only plots the analysis results of four datasets (i.e., the data files named 130, 170, 3001 and 3003) using different methods (i.e., the proposed EDPWO, AVG, STH and EMDF). The specific analysis results are shown in Figure 18, Figure 19, Figure 20 and Figure 21, respectively. As can be seen from Figure 18, due to the fact that the periodic impact is obvious in the raw outer race fault signal, the outer race fault characteristic frequency $f_{o1}$ and its harmonics can be extracted by direct envelope spectrum analysis and all morphological filtering methods, but the peak of fault frequency obtained by the proposed method is higher than other methods. As shown in Figure 19, although direct envelope spectrum analysis, AVG, STH and EMDF, can extract the inner race fault characteristic frequency $f_{i2}$, their extracted fault frequency amplitude is not as obvious as the proposed method. However, in Figure 20 and Figure 21, whether the direct spectral analysis or different morphological filtering algorithms (i.e., the proposed method, AVG, STH and EMDF), the bearing inner race fault characteristic frequency cannot be found in their envelope spectrum. The comparative analysis above shows that the proposed method can effectively work for a selected signal but may not work for another signal with very low SNR.

To more intuitively evaluate the performance of various methods, Table 5 lists the statistical evaluation results of various methods on all available signals from the 12 k drive end of CWRU. Of particular note is that in Table 5, √ represents that this method can work for the selected data (i.e., bearing fault features can be extracted), ✕ represents that this method cannot work for the selected data (i.e., bearing fault features cannot be extracted), and * denotes data not available. As can be seen from Table 5, except for the inner race fault data with 0.028 inches, four morphological filtering algorithms (i.e., EDPWO, AVG, STH and EMDF) can work for other selected inner race fault data. As listed in Table 5 (continued 1), due to a lot of noise and the interference of signal transmission path, except for a few sets of data (e.g., 3005, 3006, 3007 and 3008), four morphological filtering algorithms (i.e., EDPWO, AVG, STH and EMDF) cannot basically work for ball fault data. Moreover, as can be seen from Table 5 (continued 2~4), except for the outer race fault data with 0.014 inches at 6 o’clock, four morphological filtering algorithms (i.e., EDPWO, AVG, STH and EMDF) can all extract outer fault features. That is, the proposed method can work for most of the selected outer race fault data. Therefore, through this comprehensive statistical evaluation, it can be explained again that the morphological filtering method in this paper will be appropriate for most bearing fault information extraction, but it may not be applicable to noisy signals with very low SNR. That is, the morphological filtering method in this paper has advantages and disadvantages.

### 5.3. Case 3: Experimental Data from Laboratory

#### 5.3.1. Experimental Platform and Data Acquisition

**Figure 22:**
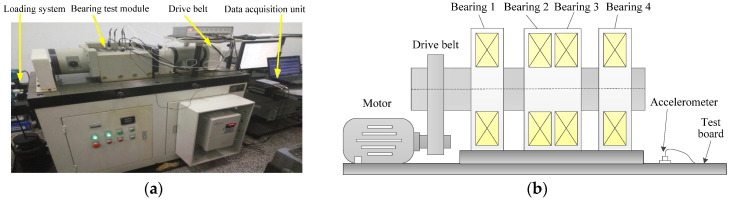
(**a**) The experimental platform; (**b**) its structure schematic drawing.

**Figure 23:**
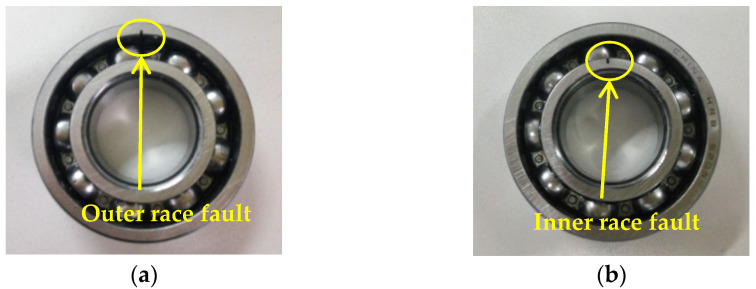
(**a**) Outer race fault bearing; (**b**) inner race fault bearing.

Experimental data from the mechanical fault diagnosis laboratory of Southeast University (SEU) is adopted to validate the effectiveness of the proposed method. Figure 22a,b respectively show the experimental platform and its structural schematic drawing, which consists of a loading system, bearing test module and data acquisition unit, and so on. The bearing test module has four bearings (i.e., bearing 1~4), where bearing 1 is the faulty bearing and the other bearings (i.e., bearing 2~4) are normal bearings. In this experiment, the local fault (see Figure 23) with a width of 0.1 mm and a depth of 0.5 mm is artificially manufactured on the inner race and outer race of a rolling bearing by using electrical discharge machining (EDM). Moreover, one accelerometer (see Figure 22b) is installed at a position far away from the faulty bearing (bearing 1) to collect the bearing’s weak fault signal. The motor speed during the experiment is about 1050 r/min (i.e., the rotating frequency $f_{r5}$ = 17.5 Hz). The sampling frequency and sampling number are set as 10,240 Hz and 5120 points, respectively. Table 6 lists the detailed specifications of the testing bearing. The bearing outer race and inner race fault frequencies are $f_{o5}$ = 62.73 Hz and $f_{i5}$ = 94.76 Hz, respectively.

#### 5.3.2. Outer Race Fault Signal Analysis and Comparative Study

**Figure 24:**
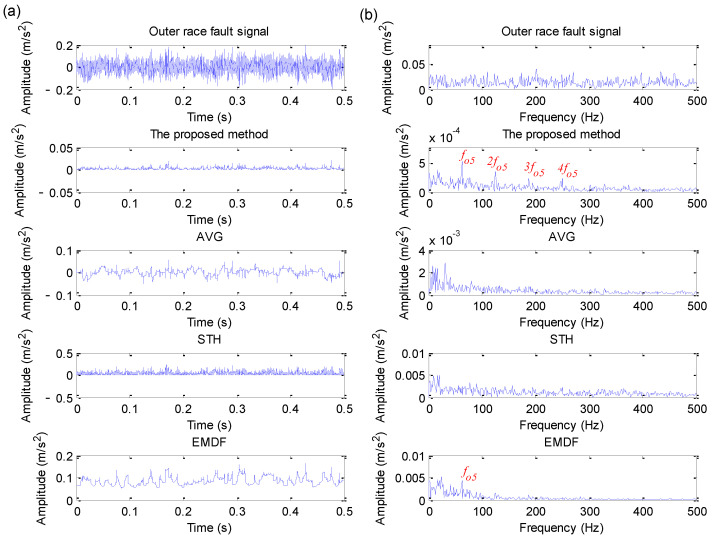
Analysis results obtained by different methods for outer race fault signal in case 3: (**a**) Time domain waveform; (**b**) its corresponding envelope spectrum.

The collected bearing outer race fault signal in case 3 is processed by using direct envelope spectrum analysis and four methods (i.e., the proposed method, AVG, STH and EMDF). Figure 24a,b show the time domain waveform and its corresponding envelope spectrum obtained by different methods for bearing outer race fault signal in case 3, respectively. As can be seen from the envelope spectrum of Figure 24b, when the direct envelope spectrum analysis and three traditional morphological filtering methods (e.g., AVG, STH and EMDF) are conducted to analyze the bearing outer race fault signal, the outer race fault frequency cannot be extracted effectively. However, when the proposed method is applied to analyze the outer race fault signal, there is an obvious peak at the outer race fault frequency of $f_{o5}$ = 62.73 Hz and its harmonics ($2f_{o5}$, $3f_{o5}$, $4f_{o5}$). Therefore, according to this comparison, it can be concluded that the proposed method has greater superiority in extracting outer race fault information.

#### 5.3.3. Inner Race Fault Signal Analysis and Comparative Study

**Figure 25:**
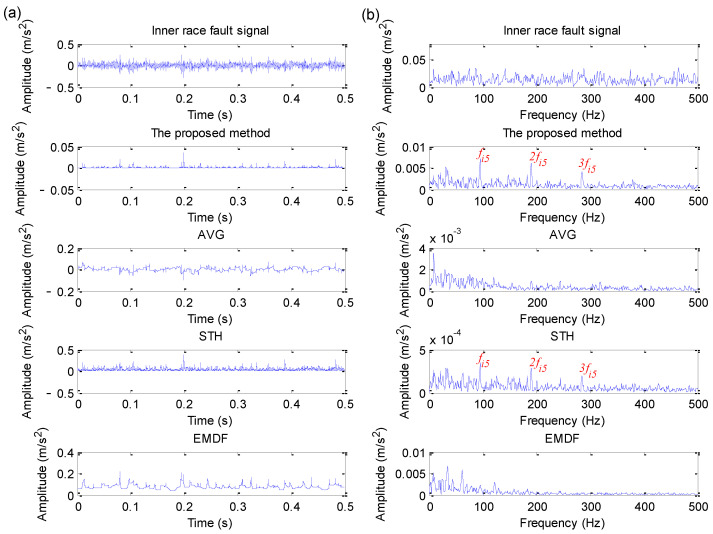
Analysis results obtained by different methods for inner race fault signal in case 3: (**a**) Time domain waveform; (**b**) its corresponding envelope spectrum.

Similarly, direct envelope spectrum analysis and four methods (i.e., the proposed method, AVG, STH and EMDF) are adopted to deal with the collected bearing inner race fault signal in case 3. Figure 25a,b show the time domain waveform and its corresponding envelope spectrum obtained by different methods for bearing inner race fault signal in case 3, respectively. It can be clearly found from the envelope spectrum of Figure 25b that the proposed method and STH operator can effectively extract bearing inner race fault frequency $f_{i5}$ = 94.76 Hz and its harmonics ($2f_{i5}$, $3f_{i5}$), but bearing inner race fault frequency is invisible in the analysis results of the other three methods (i.e., direct envelope spectrum analysis, AVG and EMDF). Therefore, in this case, the fault feature extraction capability of the proposed method is better than that of the other three methods (i.e., direct envelope spectrum analysis, AVG and EMDF). That is, the proposed method has good noise reduction performance and can be effectively applied for fault feature extraction of the bearing inner race in case 3. 

### 5.4. Discussion and Future Prospects

To further quantitatively compare the fault feature extraction abilities of different methods, we calculated three statistical evaluation indicators (i.e., FFR, kurtosis and CPU time value) obtained by various methods for simulation signals and different experimental fault data. All experiments are conducted on a computer configured with Intel(R) Core(TM) i7-9750H, CPU 2.60 GHz and RAM 8 GB. The detailed calculation results of statistical indicators of different data are shown in Table 7, Table 8, Table 9, Table 10, Table 11 and Table 12. Of particular note is, due to space limitation, this section only calculates the quantitative analysis results of eight datasets (i.e., the data files named 130, 198, 236, 237, 105, 170, 211 and 3004) for CWRU data. Whether the simulation signal or experimental signal, FFR and kurtosis value of the proposed method are obviously larger than that of the raw signal and other three morphological filtering methods (i.e., AVG, STH and EMDF). In terms of computational efficiency, the CPU time of the proposed method is higher than that of the raw signal and the other two methods (i.e., AVG and STH), but less than that of EMDF. As a whole, with the rapid development of modern high-speed computers, the computation time of the proposed method can be reduced to meet a real need. Due to the statistical indicators (i.e., FFR and kurtosis) reflecting the impact characteristics of the signal, we can reach a conclusion from the above quantitative comparison results that the bearing fault feature extraction performance of the proposed method exceeds direct envelope spectrum analysis and traditional morphological filtering methods (i.e., AVG, STH and EMDF). Moreover, due to the introduction of differential products and weighted operations in morphological filtering, the proposed EDPWO has good noise resistance and is provided with certain enhancement ability of fault characteristic information. However, most fault diagnosis methods (e.g., spectral kurtosis (SK), maximum correlated kurtosis deconvolution (MCKD), wavelet packet decomposition (WPT), empirical mode decomposition (EMD) and local mean decomposition (LMD)) have advantages and disadvantages. For instance, when the SK is adopted to process bearing fault signals, the number of decomposition levels of kurtogram demand to be set artificially. Although MCKD improved the extraction of periodic impulse-train of minimum entropy deconvolution (MED) with the help of correlated kurtosis, but its ability is closely associated with several parameters (i.e., the filtering length, the deconvolution period and the shift order of deconvolution). The performance of WPT depends largely on the selection of the appropriate wavelet basis function and decomposition level. The obstacles of EMD and LMD are the end effects and mode mixing, and their iteration conditions are not fixed absolutely. Similarly, the proposed method also has some disadvantages and is plagued by the problem of needing to know the fault frequencies in advance. Therefore, the problems existing in our proposed method and the future prospects are discussed and presented in the following content.

(1) Due to the performance of the proposed method will be significantly affected by the lower SNR, especially below the SNR of −6 dB. Therefore, in future work, in order to satisfy bearing fault feature extraction with lower SNR, we will combine the proposed method with other advanced filtering methods (e.g., blind deconvolution [34], adaptive signal decomposition [35] and sparse representation) for further enhancing bearing fault feature extraction.
(2) Considering that some prior knowledge of bearing fault characteristic frequency is required in the calculation process of FFR of our proposed method, this tends to hinder the application of the proposed method in real engineering. That is, this prior requirement is regarded as the disadvantage of our proposed method. Therefore, in our future work, to satisfy the actual requirements, we will explore some effective indexes (e.g., sparsity measure based on autocorrelation function) without prior knowledge instead of FFR to select the optimal combination parameters of the proposed EDPWO. Specifically, to improve the fault feature extraction ability of our proposed method and avoid the dependence on prior knowledge of bearing fault frequencies, we will work on a new indicator named integrated measure of sparsity-impact (IMSI) to replace FFR to select the optimal SE length of our proposed method. Moreover, the effectiveness and superiority of this IMSI index in bearing fault feature extraction will be launched and promoted steadily in the follow-up work.
(3) In this paper, the proposed method is used for analyzing bearing single faults, but its performance is unknown for multiple fault detection. Hence, in order to meet the requirements of the proposed method for synchronous intelligent online diagnosis of multi-bearing faults, the proposed EDPWO will be fused with deep learning models (e.g., deep variational auto-encoder [36], bidirectional long short-term memory [37] and deep graph convolutional network [38,39]) to automatically achieve health condition identification of different bearing fault patterns.

## 6. Conclusions

In this paper, a novel method praised as enhanced differential product weighted morphological filtering (EDPWMF) is proposed for bearing fault feature extraction, which can not only suppress noise interference effectively but also retain the detailed information of the signal. Simulation and experimental signal analysis verify the effectiveness of the proposed method in bearing fault feature extraction. The detailed contributions and innovations of this paper are as follows:

(1) An enhanced differential product weighted morphological operator (EDPWO) is presented by integrating the differential product operation and weighted operation into four basic combinations of morphological operators.
(2) To avoid the problem that EDPWO selects parameters according to artificial experience, the fault feature ratio (FFR) index is adopted to automatically determine the optimal combination of parameters (i.e., the flat SE length and the weighting correlation factors) of EDPWO.
(3) Through the analysis of simulation signals and two experimental cases, the effectiveness of the proposed method in bearing fault feature extraction is verified. In addition, compared with the traditional morphological filtering method (i.e., AVG, STH and EMDF), the proposed method can obtain the larger FFR values, which is more conducive to the extraction of bearing fault feature information.

A point worth mentioning is, to preferably conform the actual engineering needs, in future work, we will also focus on integrating morphological filters with transfer learning or meta learning to achieve health status assessment and life prediction of large-scale industrial equipment (e.g., wind turbines, high-speed trains and aero-engines).

## Figures and Tables

**Table 1:** Specification parameter of experimental bearing.

Bearing Type	Ball Diameter	Pitch Diameter	Number of Ball	Contact Angle
N205	7.5 mm	38.5 mm	12	0°

**Table 2:** Bearing fault characteristic frequency.

Rotating Frequency *f_r_*	Inner Race Fault Frequency *f_i_*	Outer Race Fault Frequency *f_o_*	Ball Fault Frequency *f_b_*	Cage Fault Frequency *f_c_*
24 Hz	172.05 Hz	115.94 Hz	59.26 Hz	9.67 Hz

**Table 3:** Size parameters of testing bearing in case 2.

Bearing Type	Ball Diameter	Pitch Diameter	Number of Ball	Contact Angle
SKF6205-2RS	7.94 mm	39.04 mm	9	0°

**Table 4:** Bearing fault characteristic frequency in case 2.

Motor Load (Hp)	Motor Speed (rpm)	Rotating Frequency	Inner Race Fault	Outer Race Fault	Ball Fault
0	1797	$f_{r1}$ = 29.95 Hz	$f_{i1}$ = 162.19 Hz	$f_{o1}$ = 107.36 Hz	$f_{b1}$ = 141.09 Hz
1	1772	$f_{r2}$ = 29.53 Hz	$f_{i2}$ = 159.93 Hz	$f_{o2}$ = 105.87 Hz	$f_{b2}$ = 139.21 Hz
2	1750	$f_{r3}$ = 29.17 Hz	$f_{i3}$ = 157.94 Hz	$f_{o3}$ = 104.56 Hz	$f_{b3}$ = 137.48 Hz
3	1730	$f_{r4}$ = 28.83 Hz	$f_{i4}$ = 156.14 Hz	$f_{o4}$ = 103.36 Hz	$f_{b4}$ = 135.92 Hz

**Table 5:** Statistical evaluation results on all available signals from 12 k drive end of CWRU.

**Fault Diameter (inches)**	**Motor Load (Hp)**	**Motor Speed (rpm)**	**Inner Race Fault Data**	**Various Methods Work or Not**			
				**EDPWO**	**AVG**	**STH**	**EMDF**
0.007	0	1797	105	√	√	√	√
	1	1772	106	√	√	√	√
	2	1750	107	√	√	√	√
	3	1730	108	√	√	√	√
0.014	0	1797	169	√	√	√	√
	1	1772	170	√	✕	√	√
	2	1750	171	√	√	√	√
	3	1730	172	√	√	√	√
0.021	0	1797	209	√	√	√	√
	1	1772	210	√	√	√	√
	2	1750	211	√	√	√	√
	3	1730	212	√	√	√	√
0.028	0	1797	3001	✕	✕	✕	✕
	1	1772	3002	✕	✕	✕	✕
	2	1750	3003	✕	✕	✕	✕
	3	1730	3004	✕	✕	✕	✕
**Continued 1**							
**Fault Diameter (inches)**	**Motor Load (Hp)**	**Motor Speed (rpm)**	**Ball Fault Data**	**Various Methods Work or Not**			
				**EDPWO**	**AVG**	**STH**	**EMDF**
0.007	0	1797	118	✕	✕	✕	✕
	1	1772	119	✕	✕	✕	✕
	2	1750	120	✕	✕	✕	✕
	3	1730	121	✕	✕	✕	✕
0.014	0	1797	185	✕	✕	✕	✕
	1	1772	186	✕	✕	✕	✕
	2	1750	187	✕	✕	✕	✕
	3	1730	188	✕	✕	✕	✕
0.021	0	1797	222	✕	✕	✕	✕
	1	1772	223	√	✕	√	✕
	2	1750	224	✕	✕	✕	✕
	3	1730	225	✕	✕	✕	✕
0.028	0	1797	3005	√	√	√	√
	1	1772	3006	√	√	√	√
	2	1750	3007	√	√	√	√
	3	1730	3008	√	√	√	√
**Continued 2**							
**Fault Diameter (inches)**	**Motor Load (Hp)**	**Motor Speed (rpm)**	**Outer Race Fault Data at 6 O’clock**	**Various Methods Work or Not**			
				**EDPWO**	**AVG**	**STH**	**EMDF**
0.007	0	1797	130	√	√	√	√
	1	1772	131	√	√	√	√
	2	1750	132	√	√	√	√
	3	1730	133	√	√	√	√
0.014	0	1797	197	✕	✕	✕	✕
	1	1772	198	√	✕	√	✕
	2	1750	199	✕	✕	✕	✕
	3	1730	200	✕	✕	✕	✕
0.021	0	1797	234	√	√	√	√
	1	1772	235	√	√	√	√
	2	1750	236	√	√	√	√
	3	1730	237	√	√	√	√
0.028	0	1797	*	*	*	*	*
	1	1772	*	*	*	*	*
	2	1750	*	*	*	*	*
	3	1730	*	*	*	*	*
**Continued 3**							
**Fault Diameter (inches)**	**Motor Load (Hp)**	**Motor Speed (rpm)**	**Outer Race Fault Data at 3 O’clock**	**Various Methods Work or Not**			
				**EDPWO**	**AVG**	**STH**	**EMDF**
0.007	0	1797	144	√	√	√	√
	1	1772	145	√	√	√	√
	2	1750	146	√	√	√	√
	3	1730	147	√	√	√	√
0.014	0	1797	*	*	*	*	*
	1	1772	*	*	*	*	*
	2	1750	*	*	*	*	*
	3	1730	*	*	*	*	*
0.021	0	1797	246	√	√	√	√
	1	1772	247	√	√	√	√
	2	1750	248	√	√	√	√
	3	1730	249	√	√	√	√
0.028	0	1797	*	*	*	*	*
	1	1772	*	*	*	*	*
	2	1750	*	*	*	*	*
	3	1730	*	*	*	*	*
**Continued 4**							
**Fault Diameter (inches)**	**Motor Load (Hp)**	**Motor Speed (rpm)**	**Outer Race Fault Data at 12 O’clock**	**Various Methods Work or Not**			
				**EDPWO**	**AVG**	**STH**	**EMDF**
0.007	0	1797	156	√	√	√	√
	1	1772	158	√	√	√	√
	2	1750	159	√	√	√	√
	3	1730	160	√	√	√	√
0.014	0	1797	*	*	*	*	*
	1	1772	*	*	*	*	*
	2	1750	*	*	*	*	*
	3	1730	*	*	*	*	*
0.021	0	1797	258	√	√	√	√
	1	1772	259	√	√	√	√
	2	1750	260	√	√	√	√
	3	1730	261	√	√	√	√
0.028	0	1797	*	*	*	*	*
	1	1772	*	*	*	*	*
	2	1750	*	*	*	*	*
	3	1730	*	*	*	*	*

Note: √ represents this method can work for the selected data, ✕ represents this method cannot work for the selected data, and * denotes data not available.

**Table 6:** Size parameters of testing bearing in case 3.

Bearing Type	Ball Diameter	Pitch Diameter	Number of Ball	Contact Angle
HRB6205	7.94 mm	39.04 mm	9	0°

**Table 7:** Quantitative comparison of FFR of different methods.

Different Methods	Simulation Signal	Case 1: Outer Race Fault Signal	Case 1: Inner Race Fault Signal
Raw signal	0.036	2.906	2.399
EDPWO	0.149	291.788	117.588
AVG	0.044	11.142	5.521
STH	0.102	20.294	11.690
EMDF	0.062	14.676	5.834

**Table 8:** Quantitative comparison of kurtosis and CPU time of different methods.

Different Methods	Simulation Signal		Case 1: Outer Race Fault Signal		Case 1: Inner Race Fault Signal	
	Kurtosis	CPU Time (s)	Kurtosis	CPU Time (s)	Kurtosis	CPU Time (s)
Raw signal	2.844	0.019	1.600	0.018	1.498	0.019
EDPWO	9.497	52.601	52.907	180.088	176.813	182.229
AVG	2.357	14.664	1.565	56.343	1.481	55.899
STH	4.908	12.721	19.716	45.333	13.669	46.500
EMDF	3.143	59.977	2.477	215.084	4.862	220.197

**Table 9:** The FFR value obtained by various methods under different CWRU fault data.

Different Methods	Case 2: 12 kHz Drive End Bearing Outer Race Fault Data at 6 O’clock				Case 2: 12 kHz Drive End Bearing Inner Race Fault Data			
	Dataset 130	Dataset 198	Dataset 236	Dataset 237	Dataset 105	Dataset 170	Dataset 211	Dataset 3004
Raw signal	0.466	0.002	0.216	0.253	0.052	0.023	0.263	0.029
EDPWO	0.635	0.006	0.344	0.405	0.135	0.054	0.353	0.049
AVG	0.049	0.002	0.051	0.072	0.034	0.007	0.042	0.012
STH	0.316	0.006	0.328	0.397	0.063	0.033	0.269	0.031
EMDF	0.443	0.004	0.278	0.322	0.061	0.039	0.304	0.031

**Table 10:** The kurtosis value obtained by various methods under different CWRU fault data.

Different Methods	Case 2: 12 kHz Drive End Bearing Outer Race Fault Data at 6 O’clock				Case 2: 12 kHz Drive End Bearing Inner Race Fault Data			
	Dataset 130	Dataset 198	Dataset 236	Dataset 237	Dataset 105	Dataset 170	Dataset 211	Dataset 3004
Raw signal	7.703	2.943	19.656	21.855	5.459	28.778	6.961	3.364
EDPWO	15.967	7.739	57.222	88.180	34.414	199.842	31.638	27.836
AVG	9.753	3.364	19.537	20.599	6.462	25.934	5.764	4.478
STH	15.617	4.816	47.430	41.569	11.447	72.077	13.899	9.052
EMDF	4.851	3.076	13.250	14.836	5.232	34.072	4.749	3.617

**Table 11:** The CPU time obtained by various methods under different CWRU fault data(s).

Different Methods	Case 2: 12 kHz Drive End Bearing Outer Race Fault Data at 6 O’clock				Case 2: 12 kHz Drive End Bearing Inner Race Fault Data			
	Dataset 130	Dataset 198	Dataset 236	Dataset 237	Dataset 105	Dataset 170	Dataset 211	Dataset 3004
Raw signal	0.018	0.019	0.019	0.018	0.018	0.018	0.019	0.019
EDPWO	90.285	93.653	94.349	92.141	92.314	88.780	92.245	92.868
AVG	30.056	30.272	30.646	30.094	30.371	29.964	30.258	30704
STH	23.568	23.221	23.283	23.358	23.584	23.262	23.305	23.130
EMDF	112.093	111.243	113.147	112.354	114.024	111.223	111.477	111.379

**Table 12:** Quantitative comparison of statistical indicators of different methods in case 3.

Different Methods	Case 3: Outer Race Fault Signal			Case 3: Inner Race Fault Signal		
	FFR	Kurtosis	CPU Time (s)	FFR	Kurtosis	CPU Time (s)
Raw signal	0.005	3.029	0.012	0.005	4.714	0.018
EDPWO	0.066	10.256	146.097	0.037	101.890	153.317
AVG	0.001	2.991	52.121	0.002	3.606	49.005
STH	0.003	4.964	41.115	0.018	14.085	39.615
EMDF	0.006	3.387	190.205	0.007	5.426	177.441

## Data Availability

The data used in this study are all owned by the research group and will not be transmitted.
